# Diversity of Mycotoxigenic *Penicillium* and Associated Mycobiota in Dry-Cured Meat (*Cecina,* León, Spain) Revealed by a Polyphasic Approach

**DOI:** 10.3390/foods15061056

**Published:** 2026-03-17

**Authors:** Daniela Cristina Solo de Zaldivar Ribeiro, Alberto Pintor-Cora, Ángel Alegría, Jesús A. Santos, Jose M. Rodríguez-Calleja, Teresa M. López-Díaz

**Affiliations:** Department of Food Hygiene and Food Technology, Veterinary Faculty, Universidad de León, Campus de Vegazana s/n, 24071 León, Spain; danicrissolo@gmail.com (D.C.S.d.Z.R.); apintc@unileon.es (A.P.-C.); a.alegria@unileon.es (Á.A.); j.santos@unileon.es (J.A.S.); jm.rcalleja@unileon.es (J.M.R.-C.)

**Keywords:** dry-cured meat, *cecina*, mycobiota, *Penicillium*, polyphasic identification, food safety

## Abstract

*Cecina de León* is a traditional Spanish dry-cured beef product whose surface, as in other similar meat products, becomes heavily colonised by fungi during ripening, raising concerns related to possible mycotoxin contamination. This study aimed to characterise the mycobiota associated with *cecina* and its production environment, with particular emphasis on mycotoxigenic *Penicillium* species. Seventy-eight *cecina* samples and 26 air samples were collected from meat-processing plants and local markets in the province of León (Spain) and analysed for fungal counts, water activity and pH. A total of 101 mould isolates and 16 yeasts were recovered, with *Penicillium* accounting for 88% of all moulds. Sixteen *Penicillium* species were identified using a polyphasic approach integrating macro- and micromorphological analysis, extrolite production, molecular markers (*BenA*, *CaM* and ITS), and MALDI-TOF MS. Mycotoxin screening by HPTLC and HPLC-PDA targeted cyclopiazonic acid, ochratoxin A, patulin, citrinin, griseofulvin and mycophenolic acid, revealing that 51% of the *Penicillium* isolates were mycotoxin producers, mainly *P. commune*. The proposed polyphasic strategy, including MALDI-TOF MS as a rapid complementary tool, offers a practical framework for the surveillance of fungal communities and mycotoxin risk in meat-processing environments.

## 1. Introduction

*Cecina* is a traditional dry-cured beef product from the region of León (Northwestern Spain), with a great economic and cultural importance [1]. It is characterised by a typical red colour, a smoked flavour, and a slightly salty taste. It has achieved PGI (Protected Geographical Indication) recognition due to its specific quality linked to geographical origin. In 2025, production achieved more than 100,000 pieces of PGI *cecina*. *Cecina de León* has recently attracted interest from international markets, such as Japan and Canada [2], reflecting its emerging recognition as a premium cured meat product beyond Spain. Its commercial relevance supports the selection of this product as a representative model for assessing quality and safety parameters in globally traded dry-cured meats.

A clearly visible layer of fungi grows throughout the entire production process, covering the *cecina* for several months. This is like what occurs in the manufacture of dry-cured ham [3]. The presence of some fungi on cured or fermented meat products contributes to their ripening and to the development of flavours typical of the product [4,5]. However, undesirable species may also grow and spoil the product or produce mycotoxins [6,7,8,9,10,11,12,13]. The mycobiota of *cecina* is not well known. Over the past 30 years, *cecina*-related research has addressed aspects such as chemistry and technology [14,15,16]. However, very few studies have focused on microbiology [17] and the potential risks associated with mycotoxigenic species.

The polyphasic identification [18,19] could be improved for a multi-method approach including MALDI-TOF MS [20,21,22]. Early detection of these microorganisms during production is essential to prevent spoilage [23]. Maintaining a high level of hygiene and controlling environmental conditions is therefore extremely important. In the food industry, environmental conditions such as temperature and relative humidity can favour the growth of spoilage fungi. Furthermore, the growth of potentially mycotoxigenic fungi increases health risks and could compromise the safety of consumption [24].

*Cecina de León* represents an excellent model of the traditional dry-curing long process with high colonisation of its surface by filamentous fungi. This study aimed to identify and characterise the mycobiota associated with *cecina* with particular emphasis on *Penicillium* species and its mycotoxin-production potential in food, to recognise the health risk and to improve control strategies.

## 2. Materials and Methods

### 2.1. Sampling

Over a two-year period, 78 *cecina* samples (17 whole pieces and 61 surface swabs) were collected from eight establishments in the province of León, Spain, including three meat processing plants (MPPs) and five local markets. *Cecina* surface samples were obtained from products aged at least four months—by which time their surfaces were fully colonised by fungi—using sterile swabs moistened with 0.05% Tween 80 and refrigerated during transport. These samples, along with environmental samples from the settling rooms, ripening rooms, and smoking chambers of the three MPPs collected across different seasons, were intended to capture microbial diversity throughout the entire production process [23,25], which has a minimum duration of seven months according to PGI *Cecina de León* specifications [14]. Air sampling (n = 26) was performed in the same locations using a MicroBio MB1 (Yakarta Meridional, Indonesia) air sampler (100 L/min) onto Glucose Chloramphenicol Agar (GCA, Scharlab, Barcelona, Spain) plates, which were incubated at 25 °C for 5 days. All surface samples were also plated on GCA and incubated under the same conditions. Additionally, a spread-plating method was applied to the 17 *cecina* pieces to determine total yeast and mould counts using the same medium and incubation parameters.

Water activity (aw) and pH of 17 *cecina* samples were analysed, using an Aqualab CX-2 (Decagon Devices Inc., Pullman, WA, USA) for aw, calibrated using distilled water and a lithium chloride standard (aw = 0.500 ± 0.003), while pH was determined using a Testo 205 portable metre (Testo SE & Co. KGaA, Titisee-Neustadt, Germany) with integrated NTC sensor. All measurements were performed in triplicate.

### 2.2. Polyphasic Approach

Due to the complexity of identifying *Penicillium* species and to avoid misidentification, we adopted the polyphasic approach proposed by Frisvad and Samson [18] and Visagie et al. [19], based on morphological characterisation, mycotoxin analysis (extrolites) and molecular identification. To enable rapid and cost-effective identification, some isolates were classified using a simple flowchart designed for this study that combined morphological characteristics with mycotoxin production. Certain species were identified through this process by considering characteristics that are highly unique and distinctive.

#### 2.2.1. Morphological Characterisation

Mould colonies were selected from each GCA plate according to the different morphological types found and three-point inoculated on MEA plates (25 °C/7 days) for identification at genus level according to Samson et al. [23]. To identify the *Penicillium* isolates at species level, the following media, prepared according to Samson et al. [23], were used: Malt Extract Agar (MEA), Yeast Extract Sucrose (YES), Creatine Sucrose (CREA) and Czapek Yeast Autolysate (CYA) (incubated at 25 °C/7 days, and also at 30 °C/7 days for CYA); MEA and Dichloran 18% glycerol agar (DG18) media were used for *Aspergillus* identification. A macroscopic and microscopic study of the *Penicillium* isolates was then carried out and acid/base production was observed on CREA [19,23,24].

#### 2.2.2. Mycotoxin Analysis

Standards of cyclopiazonic acid (CPA), patulin (PAT), citrinin (CIT), griseofulvin (GRI), mycophenolic acid (MPA), and ochratoxin A (OTA) were obtained from Sigma (Sigma-Aldrich, Merck, Madrid, Spain). Stock solutions (1 mg/mL) were prepared in methanol and stored at −20 °C. The following reference strains were included in the study: *P. commune* (CECT 20940) and *P. nordicum* (CECT 20939) [26], *P. expansum* (CECT 2275) and *P. verrucosum* (CECT 20766), which produced CPA, OTA, PAT, and CIT, respectively, under our laboratory conditions.

Initial screening for extrolite production was performed by HPTLC [18]. The full contents of MEA, CYA or YES plates (mycelium and agar), incubated at 25 °C/14 days, were extracted using a procedure adapted from Coton et al. [27]. Samples were homogenised 1:1 (*w*/*v*) in acetonitrile with 1% formic acid, ultrasonically extracted for 30 min, and centrifuged at 16,000 rcf for 5 min. Extracts were stored at −20 °C until analysis. TLC aluminium plates (Silica Gel 60 F254; Merck, Madrid, Spain) were used untreated or treated (or CPA and CIT). Plates were immersed in 10% oxalic acid in methanol for 2 min and heated at 110 °C/2 min. Extracts (10 µL) were applied using a Camag Nanomat 4 device (Muttenz, Switzerland). The mobile phase was toluene/ethyl acetate/90% formic acid (5:4:1) (TEF). After elution and drying, extrolites were visualised as follows: CPA by spraying Ehrlich reagent (violet spot under visible light); PAT by spraying 0.5% MBTH and heating at 105 °C/10 min (yellow spot under visible light); under short UV light (254 nm), MPA appeared as a violet spot; under long UV light (365 nm), OTA was observed as blue-turquoise fluorescent spots, CIT as yellow and GRI as violet-brown [18].

HPLC-PDA was used to confirm mycotoxin production in selected isolates. Extracts were filtered through 0.45 µm PTFE membranes. Analyses were conducted on a Waters Alliance HPLC (Milford, CT, USA) system (e2695) with a 2998 PDA (photodiode array) detector using an Ultrabase C18 column (150 × 4.6 mm, 5 µm). The gradient (40 min) employed Milli-Q water (A) and acetonitrile with 0.05% TFA (B) at 2 mL/min. Injection volume was 10 µL. HPLC analyses were performed at the Laboratory of Instrumental Techniques (LTI), University of León (Supplementary Information, Table S1).

#### 2.2.3. Molecular Identification

Most filamentous fungi isolates were subjected to DNA sequencing. Genomic DNA (gDNA) was extracted from approximately 200 mg of fungal mycelium grown on 4-day-old MEA plates using the NZY Plant/Fungi gDNA Isolation Kit (NZYTech, Lisbon, Portugal), in accordance with the manufacturer’s instructions.

The ITS region (Internal transcribed spacer specific to rDNA) and two nuclear genes β-tubulin (*BenA*), recommended for *Penicillium* by the International Commission of *Penicillium* and Aspergillus [28] and calmodulin (*CaM*), were amplified using the primers ITS1/ITS4, Bt2a/Bt2b, CMD5/CMD6, respectively, under the conditions described by Visagie et al. [19]. In brief, DNA was extracted from pure cultures and PCR amplification was performed. The quality of the amplified DNA fragments was assessed by electrophoresis. The amplicons were purified using the GeneMatrix PCR/DNA Clean-up Kit (EURx, Gdańsk, Poland) and sequenced using Sanger technology. The resulting sequences were aligned in GenBank using the BLAST program to identify the closest known relatives. Strain identification was based on BLASTN (v. 2.17.0+) searches of the NCBI (National Center for Biotechnology Information) database using ITS, β-tubulin and calmodulin sequences.

The phylogenetic tree was reconstructed from a sequence alignment performed using the ClustalW algorithm and the Neighbour-Joining (NJ) method implemented in MEGA v. 11© [29]. Phylogenetic confidence was assessed by the bootstrap test with 1000 replicates, applying the Kimura 2-parameter model for nucleotide substitution. In addition to the sequences obtained from the analysed strains, the dataset also included verified reference sequences commonly associated with meat products [13,30], which were retrieved from the ICPA database.

### 2.3. Yeast Identification

Yeast colonies were selected from the counting plates (colonies of each morphological type) and inoculated on MEA plates at 25 °C. On day 2, the yeasts were identified using the ID 32C biochemical test galleries in accordance with the manufacturer’s instructions, with support from the APIWEB™ database (bioMérieux España S.A.U., Madrid, Spain).

### 2.4. MALDI-TOF MS Identification

Fungal isolates were analysed by MALDI-TOF MS using a Microflex^®^ instrument (Bruker Daltonik, Bremen, Germany). Spectral matching was performed against the MBT Compass Reference Library 2023 (BDAL v. 13) and the MBT HT Filamentous Fungi IVD Module (Fungi v. 7.0). This reference library included 4320 species encompassing bacteria, yeasts, and filamentous fungi. The extended module for identifying filamentous fungi added around 1021 MSPs, covering approximately 225 species or groups, of which only 25 were *Penicillium* species. To achieve accurate species-level identification, the library was enhanced by incorporating additional main spectra projections (MSPs) for *P. commune* (n = 6) and *P. solitum* (n = 1) in accordance with the manufacturer’s protocol for MALDI-TOF MSP creation. These strains had previously been confirmed using a polyphasic identification approach [26]. It is important to note that *P. solitum* was not originally included in the Bruker database. This version of the library groups *P. camemberti*, *P. cyclopium*, and *P. commune* together.

Mould colonies cultivated on MEA plates were identified using MALDI-TOF on day 3, when the mycelium was fresh and clearly visible. Depending on the mould species and the degree of sporulation, CYA or YES plates were used instead of MEA plates. The Mycelium Transfer (MyT) procedure was performed using the MBT Biotarget 96 IVD (Bruker Daltonics, Bremen, Germany). A wooden inoculation pick was first dipped into a droplet of formic acid on the target plate and then used to sample the front mycelium of the mould. The collected biomass was then smeared into the formic acid droplet on the plate to facilitate protein liberation. After drying, a droplet of IVD HCCA (α-cyano-4-hydroxycinnamic acid) matrix solution was applied to allow protein extraction into the matrix solvent. Yeast identification was performed following the same MyT workflow [21].

### 2.5. Statistical Analysis

Total counts (moulds and yeasts) were log-transformed (log CFU/g) prior to analysis and expressed as the mean ± standard deviation (SD). The normality of distribution of the data for the physicochemical parameters (aw and pH) and the additional variables (age, number of *Penicillium* species, and number of mycotoxin-producing *Penicillium*) was assessed for normality using the Shapiro–Wilk test, and the homogeneity of variances using Levene’s test. Depending on the outcome of these tests, appropriate statistical tests were applied. Normally distributed variables were analysed using Student’s *t*-test and Pearson’s correlation coefficient, while non-normally distributed variables were examined using a Kruskal–Wallis test and Spearman’s rank correlation coefficient. The potential effects of season and origin on the studied variables were evaluated using the same approach. Multiple linear regression analysis was performed to further investigate relationships among variables. All statistical tests were conducted at a significance level of *p* < 0.05. The analyses were performed using JMP software (v18.2.2, SAS Institute Inc., Cary, NC, USA) and IBM SPSS Statistics software (v. 29, IBM Corp., Armonk, NY, USA). Data visualisation was carried out using Python Software Foundation (v. 3.11, Wilmington, DE, USA).

## 3. Results and Discussion

### 3.1. Physico-Chemical Parameters of Cecina

The water activity of the analysed samples (n = 17) was 0.89 ± 0.03 and the pH was 5.93 ± 0.21. The *cecina* samples had been aged for 273 ± 97 days. All three variables, aw, pH, and ageing time, followed a normal distribution. When comparing samples according to their origin (MPP and local market), no significant differences (*p* > 0.05) were observed for aw and pH. Pearson’s correlation analysis revealed a moderate but statistically significant negative correlation between aw and pH (r = −0.497, *p* = 0.042). Additionally, Spearman’s correlation analysis showed a significant association between ageing time and pH values (*p* < 0.05), indicating that longer ageing may influence high acidity. However, the correlation between ageing time and aw did not reach statistical significance (*p* = 0.066), although it approached the conventional significance threshold. In contrast, Molinero et al. [14] reported a statistically significant decrease (*p* < 0.05) in aw when comparing *cecina* samples aged 210, 270, and 360 days, suggesting that extended ripening may promote a progressive reduction in water activity under certain processing conditions. García et al. [17] reported that *cecina de León* at 153 days had pH 5.87 and aw 0.896. More recently, Gutiérrez et al. [31] described *cecina de León* reporting NaCl from 4 to 7%, pH 5.8–6.1, and aw 0.86 to 0.92. These authors noticed that parameters may vary by anatomical region (beef cut), ripening duration, and product form (whole cuts or slices). Overall, the physico-chemical parameters of analysed *cecina* samples were consistent with previous literature values.

### 3.2. Mycobiota Associated with Cecina and Air Samples

**Figure 1:**
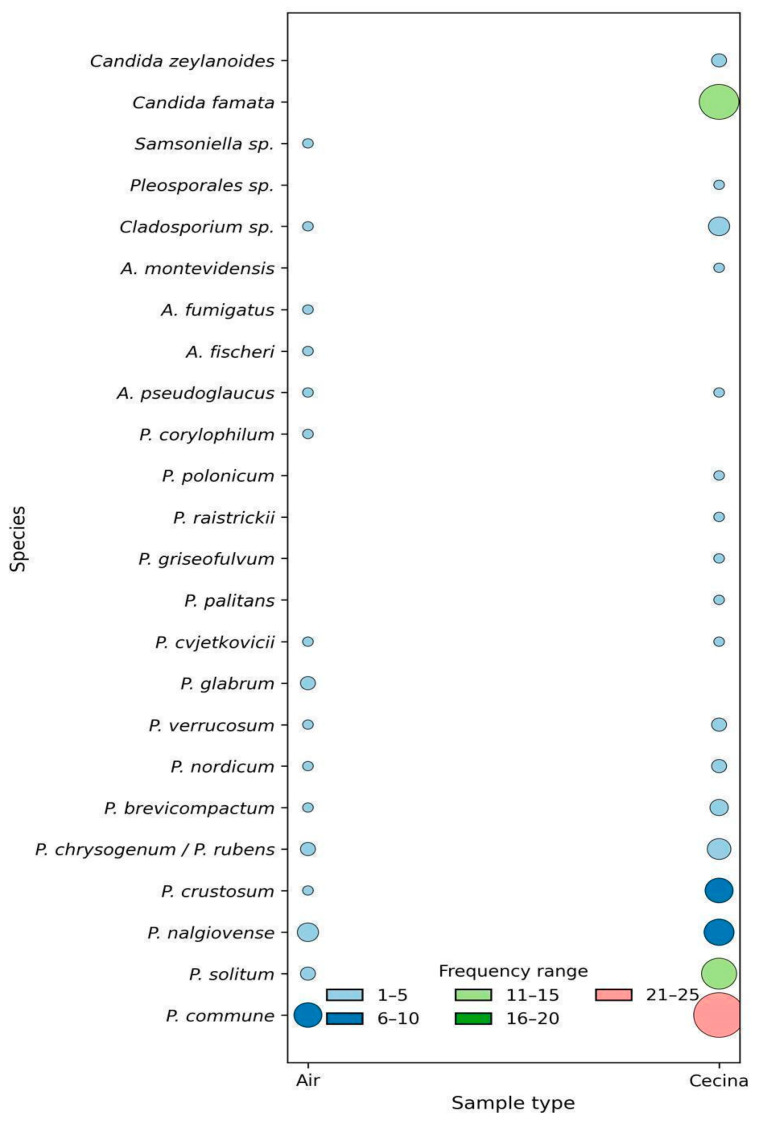
Bubble plot of fungal species isolated from *cecina* and its production environment (air) over two years (n = 117). Bubble size represents the frequency and colours indicate frequency ranges (1–5 to 21–25).

The average total count of moulds and yeasts in *cecina* was 6.58 + 0.83 log CFU/g. A total of 117 different fungi (101 moulds and 16 yeasts) were isolated from 17 samples of *cecina*, 61 *cecina* surface swabs, and 26 air plates (Table 1); 89 isolates were from *cecina* (76%), and 28 from the air (24%) (Figure 1). Following a preliminary morphological analysis, the moulds were classified at the genus level, yielding 89 isolates of *Penicillium* (88%; 66 from *cecina* and 23 from the air) and 12 isolates belonging to other genera (12%; 7 from *cecina* and 5 from the air) (Figure 1).

**Figure 2:**
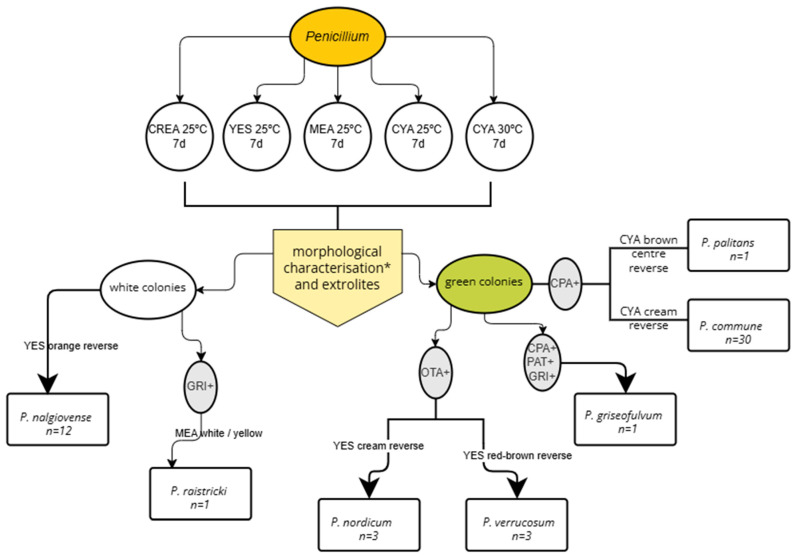
Flowchart designed for rapid phenotype-based identification of 51 *Penicillium* isolates from meat products. * Morphological characterisation included macro- and micromorphology. Media: MEA; YES; CREA, CYA. Extrolites/mycotoxins: CPA; PAT; GRI; OTA.

Sixteen different *Penicillium* species (most belonging to subgenus *Penicillium*) were identified in this study (Table 2). The polyphasic approach proposed by Frisvad and Samson [18] and Visagie et al. [19] allowed the identification of 66 strains (six strains belonging to *P. rubens/P. chrysogenum* could not be reliably distinguished, even following molecular analysis). Based on our experience, we designed a flowchart that facilitated the identification of 51 out of 89 (Figure 2), 28 of which were validated through molecular analysis. This tool therefore enabled the rapid identification based on morphology and extrolite production of seven common *Penicillium* species typically found in meat products and indoor environments. As many as 23 strains (12 *P. commune*, 10 *P. nalgiovense* and 1 *P. griseofulvum*) could be identified using only the flowchart. This integrated approach enhances the accuracy of species identification by leveraging the complementarity of multiple diagnostic methods to validate microorganism classification.

The morphological characteristics of nearly all species were consistent with the descriptions by Samson et al. [23], enabling identification through the polyphasic approach and flowchart proposed in this study (Table 2). The number of *Penicillium* species and the number of mycotoxin-producing strains showed a non-normal distribution. No significant differences (*p* > 0.05) were observed in pH, aw, or ageing time when compared with the number of *Penicillium* species. However, a statistically significant negative correlation was observed between ageing time and the number of mycotoxin-producing strains (Spearman’s ρ = −0.5701, *p* = 0.0169). This suggests that *cecina de León*, a PGI product aged for at least seven months, may contain fewer strains capable of producing mycotoxins, thereby reinforcing safety.

All *P. commune* strains (n = 30) were identified using the flowchart (18 were confirmed by molecular analysis, although other closely related species also yielded very high identity scores in BLAST) (Table 2). Despite the high similarity between *P. commune* and *P. palitans* (both CPA producers) and the comparable BLAST results, the *P. palitans* strain detected in our study was classified based on its characteristic reverse colour on CYA agar medium (creamish with a brown centre; [23]). *P. commune* was the most prevalent species both in *cecina* and air samples (Figure 1). This species can grow at low aw [24] and is commonly associated with dried meat and other foods [6,10,13,23,26,32] and has even been reported in indoor air environments [23].

*P. solitum* was the second most found species (n = 14) (Table 2), being mostly isolated from *cecina* (Figure 1). Although identification was difficult due to similarities with *P. crustosum* (morphology and extrolite production), the gene sequencing was able to distinguish them. *P. solitum* is also able to grow at low aw [24] and has been reported in European sausages and dry-cured meat products [5,13,32].

Even with molecular analysis, the distinction between *P. rubens* and *P. chrysogenum* (n = 6) was not clear (Table 2). For a more accurate identification, the study could include other extrolites, such as penicillin and PR-toxin, although their exclusive production has not been conclusively demonstrated [23,24]. *P. chrysogenum* is a ubiquitous fungus that occupies a wide range of habitats, including Spanish dry-cured hams [6,33].

*P. nordicum* (n = 3) and *P. verrucosum* (n = 3) isolates were similar (morphologies and OTA production) (Table 2), and yielded ambiguous results from the BLAST analysis. In this case, the macromorphology (in particular, the differences on the reverse colour of the colonies on YES and CYA) was a key factor that allowed identification [23]. Both species were found in *cecina* and air samples (Figure 1), and grow at low aw [23,24]. *P. nordicum* is generally associated with high-protein foods such as meat products [34], whereas *P. verrucosum* is more prevalent in cereal products and other plant sources [24], although it has also been isolated from dry-cured meat [12,35]. All tested strains of *P. nordicum* produced OTA, in agreement with other studies [11,12,18]. Similarly, the *P. verrucosum* isolates produced OTA, with none producing CIT. High salt contents favour OTA production by these species and OTA has been found in meat products [7,8,11,12,35,36,37,38].

Molecular analysis was essential to identify *P. brevicompactum* (n = 4), in conjunction with the morphological features and MPA production [23]. This species commonly occurs in European meat products such as hams, dried salami [24], and Spanish cured hams during the ripening process [39]. *P. griseofulvum* (n = 1) produced CPA, PAT and GRI, which, besides its morphological characteristics, allowed its classification using the flowchart (Figure 2). This species is often found in cereals and nuts [24], and in dry-cured meat too [36].

Two isolates were identified as *P. cvjetkovicii* by DNA sequencing, a species first described by Peterson et al. [40] forming colonies on MEA with some characteristics that did not match exactly those of our isolates (they exhibited slow growth, lacked sporulation, and displayed a dark red reverse, as well as soluble pigments on MEA). *P. cvjetkovicii* was isolated on *cecina* and air samples and has been observed in mature cheese [26].

*P. glabrum* and *P. corylophilum* (air samples) and *P. polonicum* and *P. raistrickii* (*cecina*) exhibited morphological characteristics consistent with the descriptions of Samson et al. [23] and were identified using the polyphasic approach. *P. glabrum* (formerly, *P. frequentans*) is frequently found in a variety of foodstuffs, including cured meat [24], and in indoor air [23]. *P. corylophilum* has been isolated from high-fat foods, various cereals and low-aw foods [24], from certain meat products (e.g., salami; [37]) and indoor air [23]. *P. polonicum* (formerly *P. aurantiogriseum* var. *polonicum*), a potential producer of penicillic acid and verrucosidin, has been isolated from food and indoor air [18] and from dry-cured ham [41]. Finally, *P. raistrickii* has been identified as a producer of griseofulvin, an important antibiotic that exhibits toxicity [24].

In addition to the previously mentioned wild species, *P. nalgiovense* (n = 12) was found in the environment and in *cecina* from a single processing plant and from one local market (Table 2). This species has been used as culture in meat products [29], although not specifically in *cecina*. Identification was achieved using the flowchart (Figure 2). Two strains were confirmed by gene sequencing.

Apart from *Penicillium*, other genera (*Aspergillus*, *Cladosporium*, *Pleosporales*, and *Samsoniella*) were identified by morphological and molecular analyses (Figure 1). All of these fungi are associated with meat spoilage. For example, *Cladosporium* often causes black spots, is dominant in air studies [23], and has been found in meat processing plants [5,42]. Mucorales (e.g., *Mucor*, *Rhizopus*, and *Thamnidium*) are known to produce characteristic “hairy” or “whisker-like” mycelium on meat surfaces [24]. Similarly, *Aspergillus* are well-known food spoilage fungi [23] and occur frequently in meat products and the environment. *A. pseudoglaucus* (formerly, *A. repens*) and *A. montevidensis* (formerly, *A. amstelodami*) are very common in dried meats like salami [24]. A single report has documented the presence of *Pleosporales* in pork bacon as part of the fungal diversity [43]. Species-level identification was not achieved for *Pleosporales* based on the *CaM* marker, nor for *Cladosporium* based on the *BenA* marker. In contrast, BLAST analysis of the *BenA* sequence showed 100% identity with multiple *Samsoniella* species. *Samsoniella*, a genus of growing interest due to its ecological role, bioactive compounds, and potential applications in biological control, has not been reported in industrial environments [44].

The yeasts were found in high numbers (6.83 + 0.88 log CFU/g) only in *cecina* (Table 1). *Candida famata* (14) and, to a lesser extent, *C. zeylanoides* (2) were found. *C. famata* (synonym of *Debaromyces hansenii*) is the most frequently isolated species in meat products [24,32], due to its tolerance to high sodium chloride concentrations [45]. This species exhibits lipolytic and, to a lesser extent, proteolytic activity and may contribute to colour development and flavour [46]. Furthermore, yeasts protect sausages from the adverse effects of light [47]. Regarding the mould counts, numbers were similar to those of yeasts (6.39 + 0.75 log CFU/g). Purriños et al. [48] found mould and yeast counts of 4.22 ± 1.69 log CFU/g on the surface of dry-cured lacón at the end of the manufacturing process. During the ripening phase of dry-cured ham, the action of microorganisms, primarily yeasts and moulds, plays an important role in the development of the final flavour [49].

In summary, the application of molecular identification to *Penicillium* demonstrated that *BenA* and *CaM* are useful when integrated into a polyphasic approach. To achieve reliable discrimination, most strains were assessed using combinations of two loci. In total, 49 sequences were generated for *BenA* and 47 for *CaM*. Only four of these sequences could not be assigned to a species level due to low similarity scores or ambiguous matches. In contrast, ITS proved to be markedly unreliable for species delimitation in the dataset; most ITS-based identifications (5 out of 8) were inconsistent with the polyphasic approach results, highlighting the limited resolution and poor discriminatory power of this marker for *Penicillium* identification. This is consistent with the findings of other authors [19,50].

**Figure 3:**
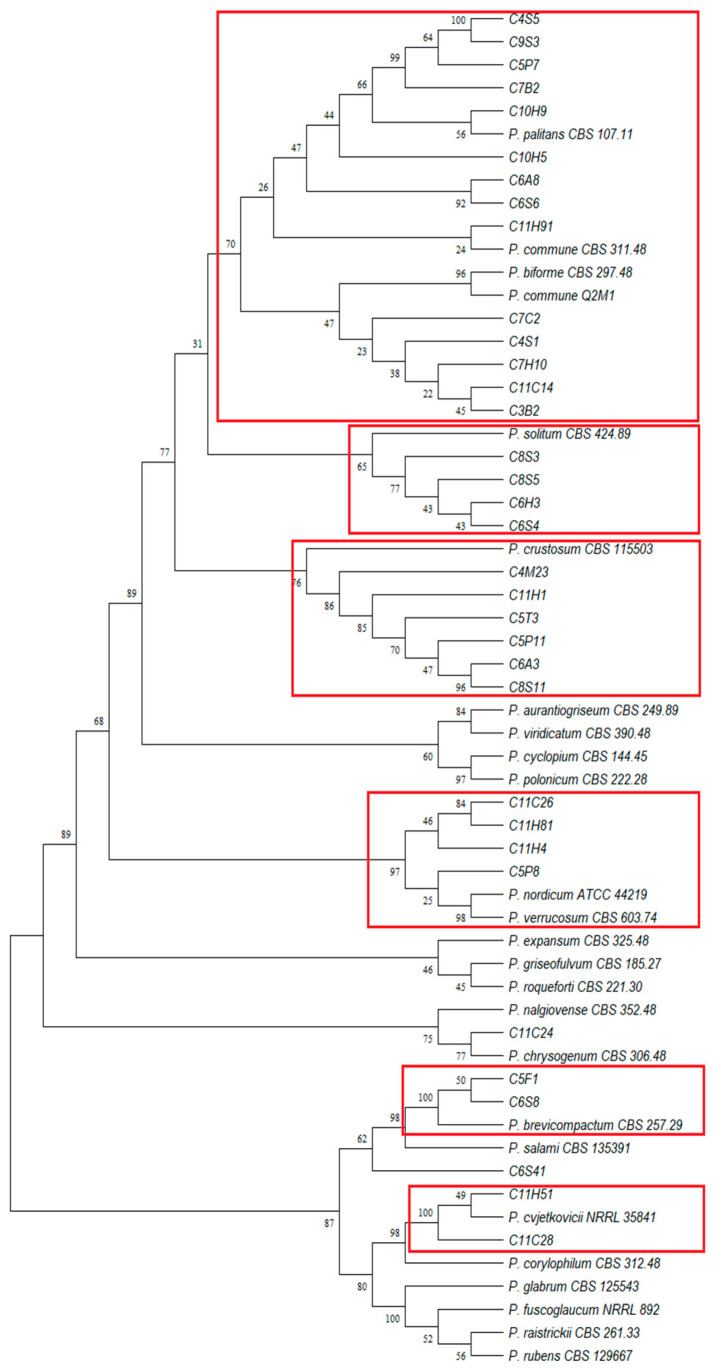
Phylogenetic relationships of *Penicillium* strains from *cecina* based on β-tubulin (*BenA*) gene sequences using the Neighbour-Joining method. Verified sequences of *Penicillium* species commonly associated with meat products are included, with bootstrap values (1000 replicates) shown on branches. The analysis included 59 nucleotide sequences (34 selected *Penicillium* strains from *cecina* and 25 verified strains). All ambiguous positions were removed using pairwise deletion, resulting in 437 positions in the final dataset. Red boxes indicate established clusters.

Phylogenetic reconstruction of *BenA* sequences from 34 selected *Penicillium* strains and the 25 verified strains yielded well-supported clades (Figure 3), consistent with the phylogenetic patterns based on *CaM* sequences (Supplementary Information, Figure S1). *P. commune* formed a well-defined clade, while other toxigenic species, such as *P. nordicum* and *P. verrucosum* appeared closely related. *P. brevicompactum*, *P. crustosum* and *P. solitum* occupied distinct clades. These phylogenetic analyses corroborated the reliability of the molecular identifications and supported the taxonomic assignments obtained through BLAST.

### 3.3. MALDI-TOF MS Identification

MALDI-TOF MS was used as a complementary tool to polyphasic identification of the isolates. A total of 73 moulds (69 *Penicillium* and 4 from other genera) and two yeasts were analysed using the MALDI-TOF MS system. Polyphasic identification (Table 2) assigned the isolates to 16 *Penicillium* species, whereas the default MALDI library could reliably distinguish only six of them. Enriching the Bruker database with in-house MSPs for *P. commune* and *P. solitum* substantially improved identification, increasing correct *P. commune* identifications from 3 to 24 out of 27 and enabling the correct classification of 6 *P. solitum* isolates. MALDI-TOF MS results for *P. verrucosum*, *P. corylophilum* and *P. glabrum* were consistent with molecular identification. Several species (e.g., *P. nordicum*, *P. rubens*, *P. griseofulvum*, *P. polonicum*) could not be resolved due to database gaps. Distinguishing between *P. chrysogenum* and *P. rubens* remained difficult with the MALDI system as *P. rubens* is absent from the Bruker database. The identification of one *A. fumigatus*, one *A. montevidensis*, and two *Cladosporium* spp. using this system was consistent with molecular results, supporting the reliability of this system for certain taxa. Regarding yeasts, only two strains were analysed by the MALDI, and both were identified as *D. hansenii*, which is consistent with the biochemical identification obtained using the ID 32C test galleries.

Patel [21] highlighted that low identification rates in MALDI-TOF MS are often linked to incomplete databases and may be improved by adding MSPs for underrepresented species. Similarly, Quéro et al. [22] showed that extending spectral libraries markedly enhances the reliability of fungal identification. In line with these findings, our results indicate that database enrichment was essential to improve the MALDI performance for *Penicillium*. Therefore, we recommend using the MALDI system in combination with morphological characteristics, extrolite profiles and, when needed, molecular markers to achieve accurate identification.

### 3.4. Food Safety Considerations

This study selected six extrolites—CPA, OTA, CIT, PAT, GRI, and MPA—considered mycotoxins associated with dry-cured meat to integrate the polyphasic approach, with CPA and OTA having already been detected in dry-cured meat in Spain [10,12,36]. Among the 89 *Penicillium* strains isolated from *cecina* and its environment, 45 (51%) could produce some of them (Table 2). Eight out of the 16 identified species were able to produce at least one mycotoxin, and another 8 were classified as non-mycotoxin producers. Moreover, *P. commune* (the dominant species in *cecina*), *P. nordicum* and *P. verrucosum* showed 100% production capacity, which suggests that investigating the presence of these mycotoxins (particularly, CPA and OTA) in *cecina* is worthwhile due to their potential health risks, with the aim of ensuring the safety of the final product, as some of these compounds might be present. In this regard, the only maximum level of mycotoxin for meat products (OTA < 1 mg/kg) is established by Italian legislation [51] merely as a recommendation, while no specific regulation exists at the European Union level, as meat products are not included in the EU mycotoxin limits [52].

## 4. Conclusions

This study provides the first comprehensive characterisation of the mycobiota associated with *cecina*, which proved to be very diverse, filling a knowledge gap regarding dry-cured meats. *P. commune* was the predominant species colonising *cecina*. Its ability to produce CPA, together with the detection of other mycotoxigenic species, highlights potential health implications and supports evidence-based decision-making in food safety management. The β-tubulin and calmodulin gene markers provided consistent species-level discrimination in conjunction with phenotype characterisation, which reinforces their role as essential components of the polyphasic taxonomy rather than as isolated criteria. Reliance on molecular data alone may result in ambiguous or low-confidence identifications. On the other hand, MALDI-TOF MS demonstrated strong potential for routine identification of *Penicillium* and other relevant fungal taxa when supported by expanded databases, reducing reliance on molecular methods in time- and cost-sensitive contexts when integrated with morphological and extrolite analyses. This combined approach enabled accurate characterisation of the fungal community in this traditional meat product. This highlights the importance of a polyphasic approach in achieving precise taxonomic resolution and providing a comprehensive assessment of potential mycotoxin-related risks. These findings provide a scientific basis for developing targeted monitoring and preventive strategies to improve quality and safety in dry-cured meat production. In particular, the implementation of environmental monitoring programmes, control of temperature and relative humidity, and the reinforcement of Good Manufacturing Practices (GMP) may help to reduce the presence of mycotoxigenic fungi throughout the production process.

## Figures and Tables

**Table 1:** Distribution of fungi isolates from *cecina* surface and production environment (air).

*Cecina*									Air				
Origin	Samples	*Penicillium*	Other Moulds	Yeasts	TOTAL Fungi				Plates	*Penicillium*	Other Moulds	Yeasts	TOTAL Fungi
MPP	73	54	5	13	72				26	23	5	0	28
Local market	5	12	2	9	11				nc	-	-	-	-
TOTAL	78 *	66	7	16	89				26	23	5	0	28

MPP: meat processing plant. nc: not collected. -: no data available. * 78 samples obtained from 17 *cecina* pieces and 61 *cecina* surface swabs.

**Table 2:** Identification and characterisation of *Penicillium* strains from *cecina* and its production environment (n = 89) using polyphasic approach (PA), flowchart (FC), and MALDI-TOF MS.

Polyphasic Approach and Flowchart									MALDI-TOF MS	
Morphological Study (n Analysed)	Extrolite ^a^ (n Positive/ n Analysed)	Gene Sequencing *BenA*, *CaM*, ITS (n Analysed)	Identification (n PA/n FC ^b^)						Identification ^c^	(n Matched Identification/ n Analysed)
*P. commune* (30)	CPA (30/30)	*P. commune/* *P. biforme/* *P. fuscoglaucum* (18)	*P. commune* (18/12)						*P. commune*	(24/27)
*P. solitum/* *P. crustosum *(14)	MPA (3/14)	*P. solitum* (14)	*P. solitum* (14)						*P. solitum*	(6/12)
*P. nalgiovense* (12)	(0/12)	*P. nalgiovense* (2)	*P. nalgiovense* (2/10)						na	na
*P. crustosum/* *P. solitum* (8)	(0/8)	*P. crustosum* (8)	*P. crustosum* (8)						*P. digitatum/* *P. italicum*	(0/8)
*P. chrysogenum/* *P. rubens* (6)	(0/6)	*P. chrysogenum/* *P. rubens* (6)	*P. chrysogenum/* *P. rubens* (6)						*P. chrysogenum*	(2/6)
*P. brevicompactum* (4)	MPA (3/4)	*P. brevicompactum* (4)	*P. brevicompactum* (4)						*P. brevicompactum*	(2/2)
*P. nordicum* (3)	OTA (3/3)	*P. nordicum/* *P. verrucosum* (3)	*P. nordicum* (3)						ni	(0/3)
*P. verrucosum* (3)	OTA (3/3)	*P. nordicum* *P. verrucosum* (3)	*P. verrucosum* (3)						*P. verrucosum*	(2/3)
*P. glabrum* (2)	(0/2)	*P. glabrum* (2)	*P. glabrum* (2)						*P. glabrum*	(2/2)
*P. cvjetkovicii* (1)	(0/1)	*P. cvjetkovicii* (2)	*P. cvjetkovicii* (2)						*P. digitatum/* *P. brevicompactum*	(0/2)
*P. palitans* (1)	CPA (1/1)	*P. commune/* *P. palitans* (1)	*P. palitans* (1)						*P. commune*	(0/1)
*P. griseofulvum* (1)	CPA, PAT, GRI (1/1)	na	*P. griseofulvum* (0/1)						ni	(0/1)
*P. raistrickii* (1)	GRI (1/1)	*P. raistrickii* (1)	*P. raistrickii* (1)						na	na
*P. polonicum* (1)	(0/1)	*P. polonicum* (1)	*P. polonicum* (1)						ni	(0/1)
*P. corylophilum* (1)	(0/1)	*P. waksmanii/* *P. corylophilum* (1)	*P. corylophilum* (1)						*P. corylophilum*	(1/1)
Total (89)	(45/89)	(66)	(66/23)							(39/69)

^a^ target mycotoxin: CPA—cyclopiazonic acid, PAT—patulin, CIT—citrinin, GRI—griseofulvin, MPA—mycophenolic acid, and OTA—ochratoxin A. ^b^ number of identified strains only through the proposed flowchart. ^c^ enhanced Bruker library. na: not analysed. ni: not included in the reference database (Bruker library).

## Data Availability

Amplicon sequencing data for the BenA and CaM loci have been deposited in the European Nucleotide Archive (ENA) under BioProject PRJEB106830. The other original contributions presented in the study are included in the article/Supplementary Materials, further inquiries can be directed to the corresponding author.

## Supplementary Materials

The following supporting information can be downloaded at: https://www.mdpi.com/article/10.3390/foods15061056/s1, Figure S1: Phylogenetic relationships of *Penicillium* strains from *cecina* based on calmodulin (CaM) gene sequences using the Neighbour-Joining method. Verified sequences of *Penicillium* species commonly associated with meat products are included, with bootstrap values (1000 replicates) shown on branches. The analysis included 69 nucleotide sequences (43 *Penicillium* strains from *cecina* and 26 verified strains). All ambiguous positions were removed using pairwise deletion, resulting in 486 positions in the final dataset. Red boxes indicate established clusters. Table S1: Performance of HPTLC and HPLC-PDA for mycotoxin (extrolite) analysis from full content of culture medium (mycelium and agar), incubated at 25 °C for 14 days.

## Abbreviations

The following abbreviations are used in this manuscript:

aw	Water activity
BenA	β-tubulin gene
CaM	Calmodulin gene
CIT	Citrinin
CPA	Cyclopiazonic acid
CREA	Creatine Sucrose Agar
CYA	Czapek Yeast Autolysate Agar
DG18	Dichloran 18% Glycerol Agar
DNA	Deoxyribonucleic Acid
ENA	European Nucleotide Archive
GCA	Glucose Chloramphenicol Agar
GRI	Griseofulvin
HPLC-PDA	High-Performance Liquid Chromatography with Photodiode Array Detector
HPTLC	High-Performance Thin-Layer Chromatography
ICPA	International Commission on *Penicillium* and *Aspergillus*
ITS	Internal Transcribed Spacer region
IVD	In Vitro Diagnostics Bruker MALDI module
JMP	Statistical analysis software SAS Institute
MALDI-TOF MS	Matrix-Assisted Laser Desorption/Ionisation Time-of-Flight Mass Spectrometry
MEA	Malt Extract Agar
ML	Maximum Likelihood if used, otherwise remove
MPA	Mycophenolic acid
MPP	Meat Processing Plant
MSP	Main Spectra Projection
MyT	Mycelium Transfer method
NCBI	National Center for Biotechnology Information
NJ	Neighbour-Joining
OTA	Ochratoxin A
PAT	Patulin
PCR	Polymerase Chain Reaction
PGI	Protected Geographical Indication
SAS	Statistical Analysis System
SD	Standard Deviation
SPSS	Statistical Package for the Social Sciences
TEF	Toluene/Ethyl acetate/Formic acid
YES	Yeast Extract Sucrose Agar
