# Supplementary material for: Diversity of Mycotoxigenic Penicillium and Associated Mycobiota in Dry-Cured Meat (Cecina, León, Spain) Revealed by a Polyphasic Approach
Source: Foods. 2026 Mar 17;15(6):1056. doi: 10.3390/foods15061056 (PMC13024893; doi:10.3390/foods15061056)

Table S1: Performance of HPTLC and HPLC-PDA for mycotoxin (extrolite) analysis from full content of culture medium (mycelium and agar), incubated at 25ºC for 14 days.

		HPTLC		HPLC-PDA		
**Mycotoxin**	**Producer strains (n)**	**Wavelength/ color spot**	**LOD (ppm)**	**t_R (min)**	**Wavelength (nm)**	**LOD (ppm)**
CPA	30	UV-VIS/violet	10.0	26.6	279	1.0
MPA	6	254 nm/violet	100.0	19	214	1.0
OTA	6	365 nm/ fluorescent blue-turquoise	1.0	23.9	254	1.0
GRI	2	365 nm/ fluorescent violet-brown	100.0	na	na	na
PAT	1	UV-VIS/yellow	10.0	1.8	276	0.25
CIT	0	365 nm/ fluorescent yellow	1.0	14.8	329	1.0

HPLC-PDA: High-Performance Liquid Chromatography with Photodiode Array Detector.
HPTLC: High-Performance Thin-Layer Chromatography.
LOD: limit of detection.
t_R: retention time.
na: not analyzed.
CPA: cyclopiazonic acid.
MPA: mycophenolic acid.
OTA: ochratoxin A.
GRI: griseofulvin.
PAT: patulin.
CIT: citrinin.
UV-VIS: ultraviolet-visible spectrum.

Figure S1. Phylogenetic relationships of *Penicillium* strains from *cecina* based on calmodulin (*CaM*) gene sequences using the Neighbor-Joining method. Verified sequences of Penicillium species commonly associated with meat products are included, with bootstrap values (1000 replicates) shown on branches. The analysis included 69 nucleotide sequences (43 *Penicillium* strains from *cecina* and 26 verified strains). All ambiguous positions were removed using pairwise deletion, resulting in 486 positions in the final dataset. Red boxes indicate established clusters.

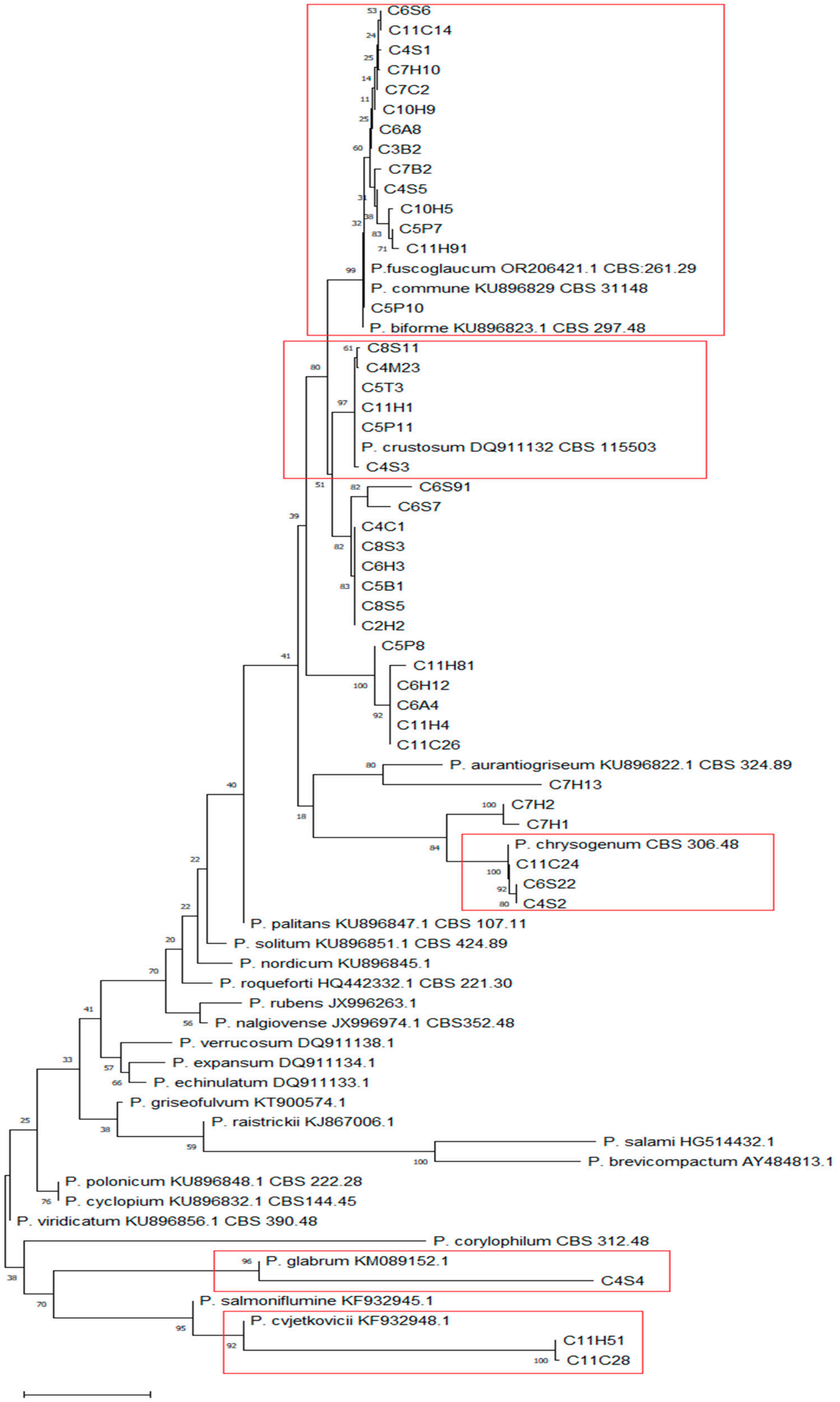

Supplement: Supplementary file 1 [file foods-15-01056-s001.zip › foods-4185335-supplementary.pdf]
